# Molecular Mechanism Underlying the Entomotoxic Effect of *Colocasia esculenta* Tuber Agglutinin against *Dysdercus cingulatus*

**DOI:** 10.3390/insects6040827

**Published:** 2015-10-07

**Authors:** Amit Roy, Sampa Das

**Affiliations:** Division of Plant Biology, Bose Institute, Centenary Campus, P1/12, CIT Scheme, VIIM, Kakurgachi, Kolkata 700054, West Bengal, India; E-Mail: amitroy_81@yahoo.co.in

**Keywords:** *Colocasia esculenta* tuber agglutinin, hemipteran pest, MALDI TOF/TOF, perimicrovillar membrane (PMM), entomotoxicity

## Abstract

*Colocasia esculenta* tuber agglutinin (CEA), a mannose binding lectin, exhibits insecticidal efficacy against different hemipteran pests. *Dysdercus cingulatus*, red cotton bug (RCB), has also shown significant susceptibility to CEA intoxication. However, the molecular basis behind such entomotoxicity of CEA has not been addressed adequately. The present study elucidates the mechanism of insecticidal efficacy of CEA against RCB. Confocal and scanning electron microscopic analyses documented CEA binding to insect midgut tissue, resulting in an alteration of perimicrovillar membrane (PMM) morphology. Internalization of CEA into insect haemolymph and ovary was documented by western blotting analyses. Ligand blot followed by mass spectrometric identification revealed the cognate binding partners of CEA as actin, ATPase and cytochrome P450. Deglycosylation and mannose inhibition assays indicated the interaction to probably be mannose mediated. Bioinformatic identification of putative glycosylation or mannosylation sites in the binding partners further supports the sugar mediated interaction. Correlating entomotoxicity of CEA with immune histological and binding assays to the insect gut contributes to a better understanding of the insecticidal potential of CEA and endorses its future biotechnological application.

## 1. Introduction

The red cotton bug (*Dysdercus cingulatus*) is a sap sucking hemipteran pest that causes considerable yield loss by feeding on the tender leaf and cotton seeds [1]. Both the adult and nymph feed gregariously on the leaves and green bolls of cotton. Consequently, the lint is stained with the crushed nymphs and deposited excreta, which negatively impacts its market value. Additionally, crushing of the bacterium *Nematospora gossypii*, which is transmitted to the boll by this bug, also contributes to lint staining [2]. Chemical insecticides are regularly employed to control such pests, but their continuous application and abuse has led to the development of resistance in many insect species. The use of transgenic plants (cotton, corn, soybean, *etc.*) containing insecticidal genes from *Bacillus thuringiensis* (Bt) that express crystal proteins to control specific target pests (lepidopteran) has reduced insecticide use, managed regional pests, and increased crop yields [3]. Unfortunately, these ICPs (insecticidal crystal protein) were documented to be ineffective against hemipteran pests [3]. Hemipteran sap sucking pests are nourished by free amino acids and amides of plant sap and thus do not have to exclusively depend on digestive enzymes for nutrition. Therefore, using protease or amylase inhibitors as antagonists [4] has turned out to be ineffective. Conversely, monocot mannose binding plant lectins (MMBL) have insecticidal activity against hemipteran, as well as other insects [5]. Lectins, basically being storage proteins, are referred to as nature’s “wonder molecule” due to their potential of appropriately switching into “defense elements” when attacked by a pest [6]. Mainly *Galanthus nivalis* agglutinin (GNA) related monocot mannose binding lectins were well recognized for their defensive role against the hemipteran group of sap sucking pests. In particular, GNA and *Allium sativum* leaf agglutinin (ASAL) show significant insecticidal efficacy, both in native as well as in transgenic expression conditions [7,8,9]. *Allium sativum* bulb lectin [ASA] was reported to induce drastic changes in color and reduces the size and weight of the red cotton bug (RCB) nymphs [2]. However, due to continuous behavioural reorientation of target pests, a single insecticidal agent is suspected to lose its entomotoxic potential with time [10]. Hence, an additional initiative was taken to extensively investigate the efficacy of other lectins against various target pests including the RCB.

*Colocasia esculenta* Linnaeus (Family: Araceae) is an annual herbaceous plant included in the high oxalate food group [11]. *C. esculenta* tuber agglutinin (CEA) belongs to *Galanthus nivalis* agglutinin (GNA)-related lectin superfamily exhibiting unique β-prism folds and binds exclusively to mannose. Each CEA monomer folds into a β- prism structure consisting of three β-sheet sub-domains (I, II and III), each formed by four anti-parallel β-strands interconnected by Ω loops. Mannose binding specificity is attained through the presence of the conserved motif QXDXNXVXY, located in each of the three sub-domains (I, II and III) [12,13].

Purification of lectin from *C. esculenta* and its anti-insect potential towards various insect pests like *Bactrocera cucurbitae* [14], *Sitophilus zeamais* adults [15], *Aphis gossypii* *and Aphis craccivora* [16] has already been documented. CEA was also found to have considerable entomotoxic effect on RCB [2,16]. However, the molecular and cellular mechanisms behind such entomotoxic effect are still unclear. Keeping this in mind, the present study was undertaken in order to unravel the molecular mechanism of the antagonistic effect of CEA on RCB.

Interestingly, confocal and scanning electron microscopic analyses revealed CEA binding to gut membrane and subsequent perimicrovillar membrane (PMM) damage in RCB nymphs fed on a CEA supplemented diet. Subsequently, ligand blot and mass spectrometric analysis were used to identify the midgut binding partners as candidate proteins responsible for CEA entomotoxicity in RCB. Thus, the present study elucidates the potential of CEA in future agriculture.

## 2. Materials and Methods

### 2.1. Plant Materials and Insects Used

Fresh tubers of *Colocasia esculenta* and nymphs of RCB were obtained from the institutional experimental farm at Madhyamgram, Kolkata according to experimental requirement. Nymphs were maintained there in sufficient numbers on young cotton plants at 26 °C and 16-h light/8-h dark cycle.

### 2.2. Extraction and Isolation of the Lectin from Colocasia sp

The lectin was isolated from 200 g of fresh *Colocasia* tuber by affinity chromatography following the protocol of Van Damme *et al.* (1995) with some modifications as described in earlier reports [17]. Total tuber protein, extracted in phosphate buffer saline (PBS, pH 7.5), was passed twice through an α-D-mannose agarose column (Sigma-Aldrich, St Louis, MO, USA) and the bound proteins were eluted by 20 mM 1,3-di amino propane. Eluted fractions from multiple collections were concentrated using membrane filter of 10 kDa cut off (Centricon, Millipore, Billerica, MA, USA), and further subjected to HPLC purification using Biosep-SEC-S2000 (300 × 7.8 mm) phenomenex column (Phenomenex, Torrace, CA, USA) monitored via Shimadzu UFLC system (Shimadzu Corporation, Kyoto, Japan) for high quality purification [18].

### 2.3. SDS-PAGE and Western Analysis

Purity of CEA was analyzed in 15% SDS-PAGE according to Laemmli (1970) [19] and through subsequent Western blotting. Western blotting was executed utilizing an anti-CEA polyclonal antibody as the primary, and anti-rabbit IgG-horseradish peroxidase conjugates as the secondary antibody [10,18].

### 2.4. Characterization of CEA through Agglutination Assays

CEA was allowed to agglutinate rabbit erythrocytes to assay its biological activity after purification as described earlier by Roy *et al.* (2014) [18].

### 2.5. Insect Bioassay on Artificial Diet

Bioassays were set up on an artificial diet formulated with some modifications [16] of the composition described by Dadd and Mittler (1966) [20]. Second instar nymphs (20/treatment/replicate) of the RCB were reared in liquid diet of 200 μL supplemented with CEA (0, 5, 10, 15, 20, 25 µg/mL each). Insect survival was recorded after every 12 h up to 72 h. Insects reared on water served as negative control. The bioassay was repeated thrice to nullify technical errors. The LC_50_ value of CEA corresponding to RCB was determined by statistical probit analysis [21].

### 2.6. Localization of CEA in RCB Midgut through Confocal Microscopy

Nymphs of RCB were reared for 48 h on artificial diet supplemented with predetermined LC_50_ of CEA. Midguts were dissected from the target insects and embedded in the water soluble embedding medium at −21 °C, and cross-sections of 15–20 µm were made using a Cryo microtome (Leica, Wetzlar, Germany). Fixation of midgut sections was performed using formaldehyde followed by extensive washing with 1× Phosphate buffer saline (PBS, pH 7.2). The sections were maintained in 2% buffered glycine followed by washing with 1× PBST (PBS with 0.2% tween20) at room temperature. Overnight blocking of sections was carried out in 2% buffered bovine serum albumin (BSA, Sigma-Aldrich) at 4 °C before washing in 1× PBS (pH 7.2). Midgut sections were then incubated with anti-CEA antibody (1:1000) for 2 h, followed by washing with 1× PBST and incubation with anti-rabbit IgG-Fluorescein isothiocyanate (FITC, Sigma-Aldrich) conjugate (1:2000) for 2 h at room temperature. The sections were then mounted in Keiser’s albumin and observed under a TCS SP5 confocal laser scanning microscope (Leica, Germany) using the excitation and emission ranges suited for FITC. Identical experimental setups were made for control insects reared on artificial diet without CEA.

### 2.7. Detection of CEA in Midgut, Haemolymph and Ovary Extract of RCB

Midgut and ovaries from 30–50 second instar nymphs of RCB, fed with either CEA supplemented diet or heat denatured CEA supplemented diet or control diet (no lectin), were dissected out, washed and crushed in liquid nitrogen. Total protein was extracted with multi detergent extraction followed by the trichloroacetic acid (TCA)/acetone precipitation method as described by Cilia *et al.* (2009) [22]. Similarly, haemolymph was extracted at intervals by cutting of insect legs as described by Feder *et al.* (1998) [23]. All samples were finally resolved in 15% SDS-PAGE followed by western blotting with anti-CEA antibody as described earlier. Samples collected from RCB, fed with control diet and heat denatured CEA supplemented diet, served as negative controls of the experiment.

### 2.8. Scanning Electron Microscopic (SEM) Analysis with the Midgut of RCB

Reared insects (RCB, second instar nymphs) were divided into three groups based on their diet. Nymphs having an artificial diet supplemented with CEA (LC_50_ dose) were considered “treated”. Other nymphs, fed only with artificial diet, were taken as positive control whereas the starved ones served as the negative control. Adult insects, 30 h, post-fed when the PMM was reported to reach its maxima [24], were dissected to isolate the midgut and the tissue samples were fixed in 3% glutaraldehyde, 4% paraformaldehyde in 0.1 M cacodylate buffer, pH 7.2. After primary fixation, the tissue samples were post fixed in 1% osmium tetroxide and then dehydrated through an acetone series before the critical point drying. Samples were then coated with gold and examined under FEI-QUANTA200 MK2 scanning electron microscope. Each set of experiments was repeated thrice to avoid technical errors.

### 2.9. Ligand Blot Assay

Total protein of RCB midgut was purified according to the protocol provided by Bandopadhyay *et al.* (2001) and Bayyareddy *et al.* (2009) [25,26]. 20 µg of total midgut protein resolved in 12% SDS-PAGE was electrophoretically transferred to a Hybond C membrane (GE Healthcare Life Sciences, Pittsburgh, PA, USA) using Hoefer (Hoefer Inc., Holliston, MA, USA) electroblot apparatus. The membrane was transiently stained with Ponceau S (Sigma-Aldrich) in order to confirm the successful transfer of proteins. Blocking of the membrane was made with 5% non-fat milk (Merck Millipore, Mumbai, India) dissolved in 1× PBST at 37 °C for 1 h. The membrane was then incubated with the predetermined LC_50_ dose of CEA for 2 h following extensive washing with 1× PBST. The membrane was then incubated with anti CEA antibody (1:8000) for 1 h at 37 °C. Further probing of the secondary antibody was performed with anti rabbit IgG HRP conjugate (1:20,000) and developed using ECL western blotting detection kit (GE Healthcare Life Sciences) on KODAK X-ray film (Eastman Kodak Company, Rochester, NY,USA).

### 2.10. Characterization of CEA Binding Proteins

#### 2.10.1. Carbohydrate Specific Staining

The total midgut protein fraction from second instar nymphs of RCB dissolved in 1% sodium deoxycholate was stained specifically for covalently bound oligosaccharides, as described by Moller *et al.* (1996) [27]. This method calls for periodic acid oxidation of the fixed proteins in SDS-PAGE gels, staining with Alcian Blue, and subsequent silver enhancement staining at high temperatures to specifically stain the oligosaccharides.

#### 2.10.2. Deglycosylation and Mannose Inhibition Assay

Total midgut protein (20 µg) from second instar nymphs of RCB was subjected to deglycosylation [28], using the *N*-Glycosidase F deglycosylation kit (Roche, Basel, Switzerland) according to the protocol described in the kit manual. The deglycosylated sample was resolved in 12% SDS-PAGE gel and ligand blot analysis was carried out. The similar ligand assay was also performed independently with 1 M α*-*D-mannose saturated CEA as described by Mondal *et al.* (2012) [29].

### 2.11. Two-Dimensional Gel Analyses of Total Midgut Protein Followed by Ligand Blot Assay

One-hundred-twenty µg of total midgut protein from second instar nymphs of RCB was resolved in two-dimensional gel electrophoresis and subsequently transported to a Hybond C membrane using Hoefer electroblot apparatus. Ligand blot analysis was performed using the same protocol as described earlier.

### 2.12. MALDI TOF/TOF Analyses

For mass spectrometry analysis, identified interactive protein spots were excised from 2D protein gels, and in-gel digestion was completed as described by Shevchenko *et al.* (2007) [30], with minor modifications. Proteins were digested in gel using sequencing grade modified porcine trypsin (Promega, Madison, WI, USA) and were extracted using 25% acetonitrile and 1% trifluoroacetic acid. 1 µL of sample and matrix (α-cyano-4-hydroxy cinnamic acid, HCCA) was loaded onto an Anchor Chip MALDI Plate (Bruker Daltonics GmbH, Bremen, Germany). Mass spectra were acquired on an Autoflex II MALDI TOF/TOF (Bruker Daltonics GmbH) mass spectrometer equipped with a pulsed nitrogen laser (λ-337 nm, 50 Hz). The spectra were then analyzed with Flex Analysis Software (version 2.4, Bruker Daltonics GmbH, Bremen, Germany). The processed spectra were searched using MS Biotools (version 3.0, Bruker Daltonics GmbH, Bremen, Germany) program, against the taxonomy of other metazoa in the MSDB 20060831 (3,239,079 sequences; 1,079,594,700 residues)/NCBI nr 20140323 (38,032,689 sequences; 13,525,028,931 residues) using MASCOT search engine (http://www.matrixscience.com; version 2.2). The peptide mass fingerprinting (PMF) parameters comprised peptide mass tolerance (≤100 ppm); proteolytic enzyme (trypsin); variable modification (oxidation, Met); global modification (carbamidomethyl, Cys); peptide charge state (1+) and maximum missed cleavage “one”. The significance threshold was set to a minimum of 95% (*p* ≤ 0*.*05). The criteria employed to accept protein identification by PMF were based on the percentage of the sequence coverage, molecular weight search (MOWSE) score, and a match of a minimum of five peptides. MS/MS was employed to validate the PMF identification with matching peptides, setting a threshold of a minimum of two peptide matches for confirming the protein identification [31].

### 2.13. Immunoprecipitation of Binding Partners and Subsequent Ligand Blot Analyses 

Five-hundred µg of midgut protein fraction solubilised in 1% sodium deoxycholate was incubated in gentle rocking condition at 4 °C with 5 µL of anti-ATPase and anti-actin antibody (Pierce, Thermo Scientific, Rockford, IL, USA). Fifty µL of 30% Protein A sepharose bid slurry (Sigma-Aldrich) in 20 mM PBS (pH 7.2) was added to both mixtures and maintained at 4 °C for 3 h. Mixtures were then centrifuged at 500× *g* for 3 min to remove the unbound proteins. The pellets containing Protein A beads with bound proteins were washed a further five times with 20 mM PBS (pH 7.5). The bound proteins were dissociated from both of the mixtures by boiling in 20 µL Laemmli buffer and separated in 15% SDS-PAGE. The Ligand blot assay was followed by challenging the bead dissociated protein fractions with CEA, same as described earlier. A replicate without anti-ATPase or anti-actin antibody served as the negative control.

## 3. Results

### 3.1. Purification, Characterization, and Monitoring of Insecticidal Efficacy of CEA on RCB

*C. esculenta* tuber lectin, purified through affinity chromatography, when subjected to 15% SDS-PAGE and western blot analysis showed a single band at ~12 kDa (Supplementary Figure S1). Agglutination assay demonstrated the lectin activity of the purified CEA [2,16]. Entomotoxicity of CEA was recorded at 12 h intervals during artificial diet based bioassay on RCB. It was clear that the toxic effect of CEA increased subsequently with increasing dose over a period of 72 h (Supplementary Figure S2). The LC_50_ value of CEA, calculated by Probit analysis against RCB, was 15.64 ± 0.268 μg/mL (Supplementary Table S1), which was comparable with earlier observations [2,16].

### 3.2. Localization and Internalization of CEA in RCB Midgut

**Figure 1:**
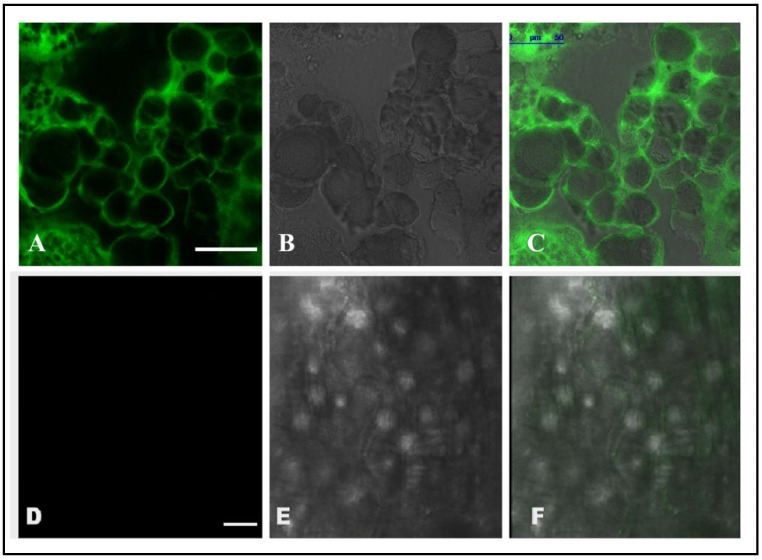
Confocal microscopic images showing the binding of CEA to the midgut epithelial cells of RCB. (**A**)—Fluorescence image showing binding of CEA to midgut epithelial cells of insects fed with CEA supplemented diet; (**B**)—Phase contrast image; (**C**)—Merged image of (**A**) and (**B**); (**D**)—Negative control where gut of control diet fed insects was used for analysis; (**E**)—Phase contrast image; (**F**)—Merged image of (**D**,**E**). (Bar indicates 50 μm).

**Figure 2:**
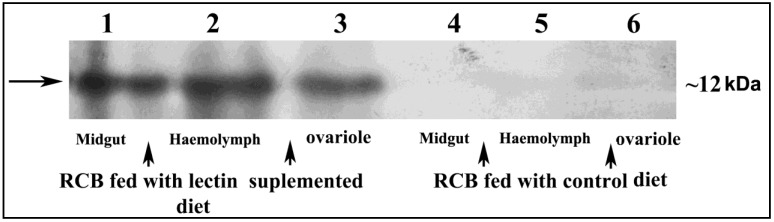
Western blotting showing the presence of CEA in the midgut, haemolymph and ovary of the lectin supplemented diet fed RCB. Lane 1, 2 and 3: total protein extract (30 µg) from lectin fed RCB midgut, haemolymph and ovary showing CEA band at ~12 kDa. Lane 4, 5 and 6: total protein extract (30 µg) from control diet (without lectin) fed RCB midgut, haemolymph and ovary showing no CEA band represented as negative control.

Confocal microscopic analysis was performed with the dissected midgut of CEA fed RCB. Obtained images documented significant FITC fluorescence in the midgut epithelial cell surface of the RCB, fed with CEA (Figure 1A–C), suggesting a specific binding of CEA to the epithelial cells of the insect midgut. In the control experiment, only diet fed RCB midguts were analysed and no such fluorescence was observed (Figure 1D–F). Moreover, CEA was detected in midgut, haemolymph and ovary extract of CEA fed RCB after Western blotting (Figure 2). The possibility of detection of non-binding CEA from diet was estimated by feeding the insect with heat denatured CEA. However, no CEA was detected in the midgut, ovary and haemolymph of RCB fed with heat denatured CEA (Supplementary Figure S3).

### 3.3. Scanning Electron Microscopy Reveals Alteration in Midgut Morphology

**Figure 3:**
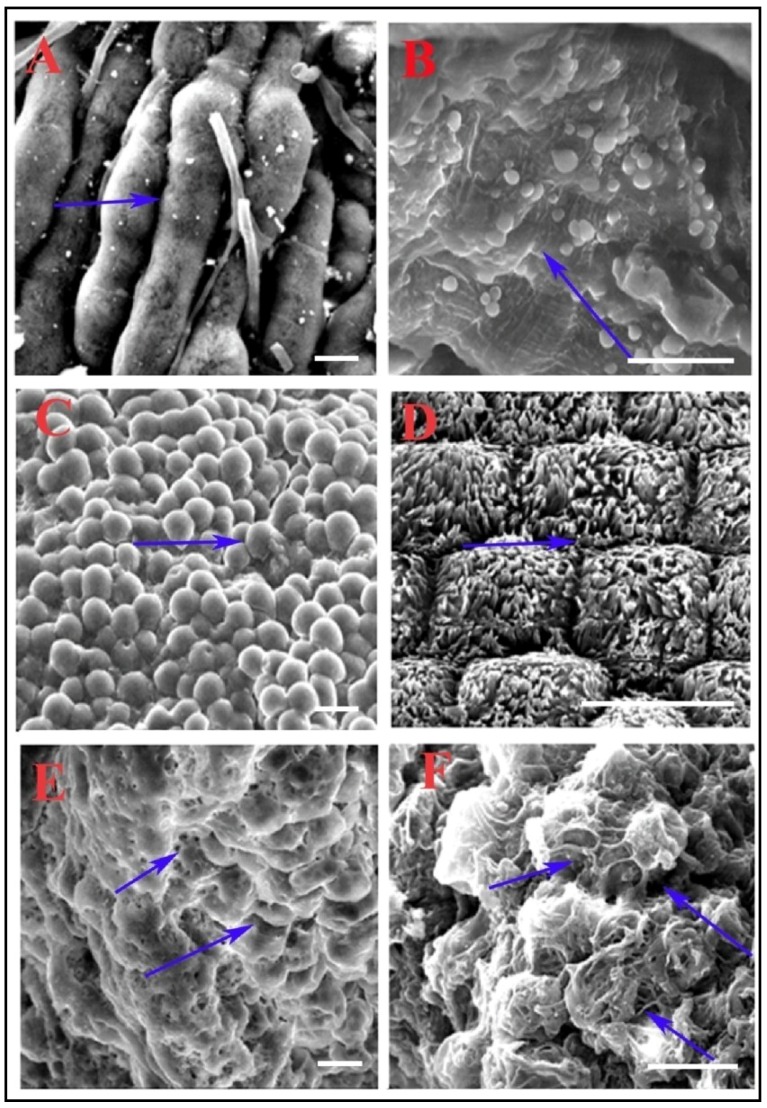
Scanning electron microscopic analysis showing morphological abnormality in CEA supplemented diet fed insect midgut epithelial cells of RCB. (**A**,**B**) showing the normal midgut morphology of well-fed insect gut; (**C**,**D**) showing the morphology of starved insect midgut; (**E**,**F**) showing the abnormal morphology of lectin supplemented diet fed insect midgut. (Bar- 800× magnification).

To observe the effect of CEA feeding on the midgut epithelial cell morphology (more specifically the PMM morphology), scanning electron microscopic analysis was performed, and midguts from 30 h post CEA fed RCB were dissected and analyzed. In control insects (without lectin fed), midgut cells were difficult to differentiate due to the masking of the well-developed PMM (Figure 3A,B), whereas in the starved insects, the phenomenon was exactly reversed. Each of the midgut cells appeared to be clearly distinguishable (Figure 3C,D) due to no PMM. On the contrary, CEA fed insect midgut cells had less well-developed PMM (Figure 3E), and substantial degradation of PMM due to toxin action (Figure 3F). Individual midgut cells could be easily spotted due to reduced cellular covering (Figure 3E,F).

### 3.4. Ligand Blot Analysis Highlighted the Midgut Binding Partners

**Figure 4:**
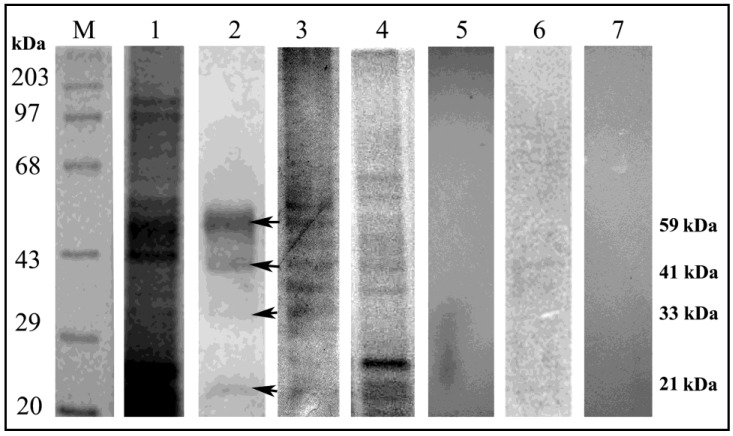
Identification and characterization of RCB midgut binding partners. Lane M: Standard protein molecular weight marker; Lane 1: SDS-PAGE (12%) profile showing Coomassie stained total midgut protein of RCB; Lane 2: Ligand blot analysis of total midgut protein of RCB showing multiple binding partners of CEA; Lane 3: Glycoprotein specific staining of total midgut protein of RCB demonstrating the glycoprotein nature of the binding partners; Lane 4: Total midgut protein profile of RCB (Coomassie stained) after deglycosylation; Lane 5: Ligand blot assay with deglycosylated midgut protein; Lane 6: Ligand blot assay of the total midgut protein using CEA presaturated with α-D-mannose; Lane 7: Negative control: Ligand blot of total midgut protein without CEA probe. (Arrows indicate midgut binding partners).

Ligand blot analysis was performed to detect putative interactive partners from the RCB midgut. Interacting proteins, predominantly in the range of 30–60 kDa, were detected (Figure 4, Lane 2).

### 3.5. Characterization of Binding Partners

Glycoprotein-specific staining demonstrated the glycoprotein nature of the ligand positive interactive proteins (Figure 4, Lane 3). Deglycosylation of total midgut protein diminished the interaction of CEA with its binding partners, highlighting the contribution of glycan moiety towards the interaction (Figure 4, Lane 5). Moreover, a ligand blot assay with mannose presaturated CEA could hardly detect any interactive proteins, thereby reiterating a mannose mediated interaction between CEA and its insect binding partners (Figure 4, Lane 6).

### 3.6. Mass Spectrometric Identification of Binding Partners from RCB

**Figure 5:**
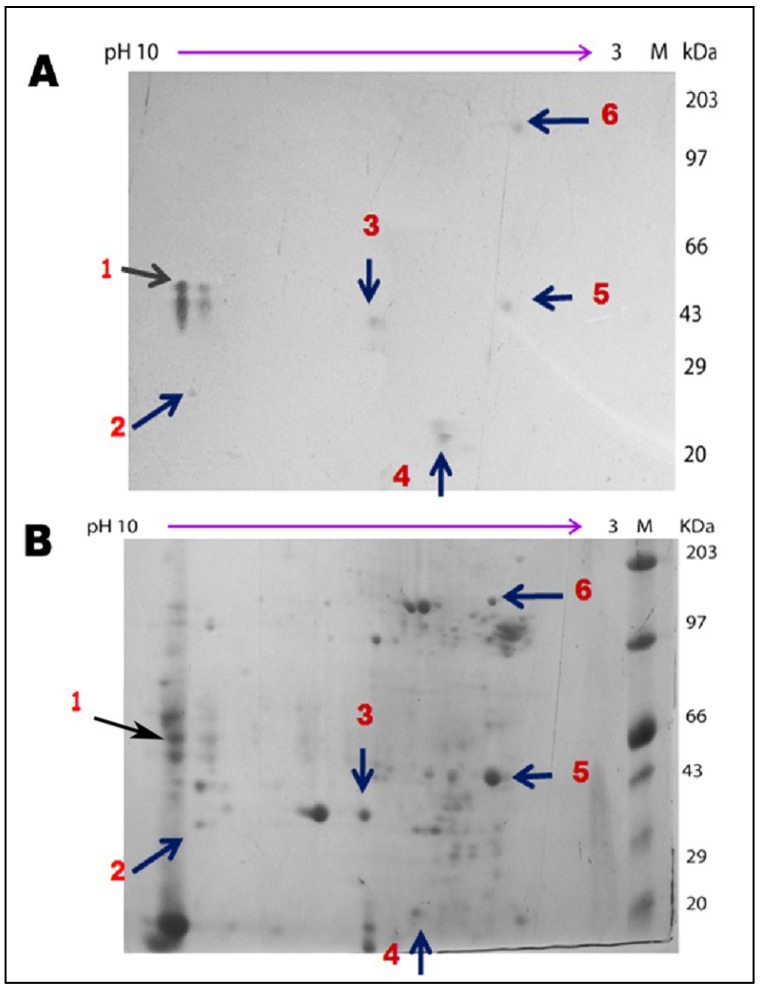
Ligand blot assay of total midgut protein fraction of RCB separated in 2D gel electrophoresis and subsequently challenged with CEA. (**A**) Ligand blot analysis of total midgut protein of RCB detected putative binding partners of CEA; (**B**) Total midgut protein of RCB stained with Coomassie (arrows indicated only those binding partners which were successfully identified in PMF).

Two dimensional gel electrophoresis analysis of the total midgut protein of RCB was performed to obtain well separated and highly concentrated protein spots. Subsequent ligand blot assay revealed the interactive protein spots (Figure 5A, B), which were successfully identified by peptide mass finger-printing technique (Table 1). Detection of three out of six identified ligand positive protein spots, namely actin, ATPase and cytochrome p450 (E class group I family) were successfully validated through MS/MS analysis and they were hypothesized to act as intra cellular binding partners of CEA (Table 1, Supplementary Figure S4). Interestingly, no extracellular membrane protein was identified as binding partner may be due to the limited sensitivity of the methods used or insufficient amount of sequence information for RCB in the database. However, ATPase may have membrane localization (membrane associated proton ATPase).

**Table 1:** Potential binding partners of CEA identified from MALDI-TOF MS and MS/MS analysis.

Spot No	Method of Identification	Accession (NCBI nr/MSDB Database)	Protein Name/Organism	Predicted kDa/pI	Observed kDa/pI	MOWSE Score: PMF/MS-MS	No of Peptide Match/Coverage (%)
1 *	PMF and MS/MS	Q5XUA1_9HEMI	ATP synthase α subunit/Brown citrus aphid	59/9.14	58/8.9	169/232	14/25%
2	PMF	Q1HPZ3_BOMMO	Inorganic phosphate carrier/Silk moth	35/9.3	33/8.7	87	6/20%
3 *	PMF and MS/MS	Q16E72_AEDAE	Cytochrome P450/Yellow fever mosquito	48/6.4	44/6.3	118/182	10/23%
4	PMF	Q17LH0_AEDAE	RNA binding protein/Yellow fever mosquito	15/5.7	19/5.3	120	8/33%
5 *	PMF and MS/MS	Q16VS2_AEDAE	Actin/Yellow fever mosquito	41/5.3	43/5.1	99/312	9/25%
6	PMF	GI:170040984	26s proteasome non ATPase regulatory subunit 1/*Culex* mosquito	112/5.5	110/5.0	106	10/14%

* Identification by PMF was successfully validated by MS/MS.

### 3.7. Authentication of Mass Spectrometric Identification of the Binding Partners

Immunoprecipitation analyses with anti actin and anti-ATP synthase antibody, followed by ligand blot analyses with CEA, highlighted identified bands obtained after initial ligand blot analysis, validating the MALDI TOF/TOF result (Supplementary Figure S5). Moreover, insect fed with diet supplemented with anti-actin antibodies significantly reduced CEA binding to midgut *in vivo* (Supplementary Figure S6).

## 4. Discussion

During the last two decades, the search for potential biological pesticides has focused on plant lectins [32,33,34,35,36]. Interestingly, monocot mannose binding plant lectins have been recognized as a broader potential biological insecticide that affects both the hemipteran and lepidopteran pests [32,37,38,39]. CEA belongs to MMBL superfamily with significant insecticidal activity against RCB [2]. Our present experiments with CEA demonstrated the basis behind such entomotoxicity.

### 4.1. CEA Binding to RCB Gut Membrane and its Internalization: the Crucial Factor for Toxicity

The insecticidal efficacy of CEA inspired a further in depth inquiry into the mode of action of the protein. Previous reports on plant-pest interaction have put forth several mechanisms of inducible plant defenses against herbivory, broadly categorized as surveillance, followed by signal transduction, and the mounting of defense [40]. Inducible plant defenses are classified as either direct or indirect, with the former being subjectively more relevant for host defense. Anti-nutrition and toxicity are the two key determinants of direct inducible defense [41]. Lectins, well established as potential insecticidal toxins, are assumed to cause detrimental effects when bound to insect midgut epithelial cells [25]. It has also been well documented that the insect midgut is the hotspot for interaction of any foreign proteins in the insect body. Hence, whatever the mechanism of activity of lectins, binding of the protein to the insect midgut plays a key role in the development of resistance against herbivory [42,43,44]. In the present study, confocal microscopy analyses revealed the binding of CEA with RCB midgut, which can be considered as the key event behind induction of entomotoxicity.

Moreover, localization of CEA in the midgut, haemolymph and ovary of CEA supplemented diet fed RCB indicated the internalization of the lectin through midgut epithelial cells as evident in the case of other lectins like GNA, ASAL, and *Hippeastrum* *hybrid* agglutinin (HHA) [8,38,39]. This finding indicates the possibility of having intracellular targets of CEA, which may contribute significantly to entomotoxicity. Interestingly, absence of CEA in the haemolymph of RCB fed with heat denatured CEA points to the importance of functional integrity for internalization. A similar observation was documented in case of GNA and Con-A (*Canavalia ensiformis*) lectins by Fitches *et al.* (2001) where both lectins failed to reach the haemolymph of tomato moth larvae, *Lacanobia oleracea,* in denatured condition [45].

Insecticidal lectins have long been reported to induce loss of fecundity in their target insects [8,43,45,46]. Although, over the years, different lectins including CEA have also been successfully identified from the ovary tissues of their target insects ([8,18,43,45] and present study), the exact molecular mechanism underlying the loss of fecundity is still unknown and demands further in depth investigation.

### 4.2. CEA Alters Midgut Perimicrovillar Membrane Morphology

Like many other hemipteran insects, the RCB possesses a special structure called PMM, a unique extra-cellular lipoprotein membrane covering the microvilli of midgut cells. Interestingly, in the RCB, this membrane (PMM) shows biphasic formation after food consumption. Based on the pattern of PMM formation, it can be concluded that PMM reaches its maximum development at 30 h after feeding [24]. CEA was found not only bound to the midgut; it also induced alteration in the midgut morphology as observed after SEM analysis, generating a circumstantial stress in comparison to the starved state of the RCB. A similar type of midgut membrane damage induced by plant lectins was also reported earlier in the lepidopteran insect, *Spodoptera littoralis* [5]. Interestingly, such membrane damaging potential of plant derived lectins may aid in its integration into insect midgut cells, and the induction of toxicity [47].

### 4.3. Sugar Mediated Interaction with Midgut Proteins

MMBL superfamily lectins have been documented to possess mannose or sugar mediated interactions with their cognate binding partners from the insect midgut [18,29]. CEA, being a member of the MMBL superfamily, also demonstrated analogous sugar mediated interaction with midgut binding partners of RCB as observed after deglycosylation and mannose inhibition assay. It is worth mentioning in this context that considerable reduction of CEA entomotoxicity was observed when RCB nymphs were fed with mannose presaturated CEA supplemented artificial diet (Supplementary Figure S8) showing the negative impact of the presaturation of binding pockets by mannose in CEA and subsequent inhibition of the interaction with binding partners. Thus, present finding endorses the importance of CEA binding to ATP synthase, actin and cytochrome P450 for exerting entomotoxicity in RCB.

Studies on pest behaviour suggested that the uptake of phloem or seed sap rich in free sugars appeared to be difficult for hemipterans, probably due to their low size-to-volume ratio. The high sugar content of phloem exerted an osmotic pressure on the insect body. To overcome this obstacle, hemipterans transform these free sugars into complex, long chain oligosaccharides, thus preventing loss of body water to the gut during feeding [48]. Plant lectins (including CEA) presumably functioned as opportunists and took part in sugar mediated interactions with insect gut membrane resulting in the disruption of cellular osmotic stoichiometry. Gel based detection of glycoprotein nature of the binding partners *i.e.*, ATP synthase, actin and Cytochrome P450 and the bioinformatic recognition of the putative glycosylation and mannosylation sites in these proteins further support the sugar mediated interaction (Figure 4, Supplementary Figure S7).

### 4.4. Mechanism of CEA Entomotoxicity

Since the hemipterans rely mostly on free amino acids for their nutrition rather than on digestive enzymes like other lepidopteran, dipteran or coleopteran classes of insects, the possibility of CEA binding preferentially to such sparingly available digestive enzymes can be statistically ruled out. Previous reports suggested that ASAL, a MMBL superfamily lectin, interacts with a chaperonin like protein symbionin (SymL), a GroEL homologue, encoded by the mutualistic *Buchnera aphidicola* that are known to reside in the gut of *L. erysimi.* *B.aphidicola* supplies aphid host with essential amino acids [28]. Earlier reports have also shown the efficiency of ASAL in the competition against the read through domain (RTD) of pathogenic viruses by binding with SymL, thus indirectly obstructing viral transmission [28]. However, in the present study, identification of CEA binding partners like actin, ATPase and Cyt P450 from RCB midgut indicates a completely different strategy for exerting entomotoxicity of plant lectins.

Interestingly, actin and ATPase were previously reported as insect midgut binding partners of different cry proteins [25,49,50]. This observed similarity may be ascribable to the fact that domain II and domain III of Cry proteins have lectin like folds which actively participate in the interaction with the receptors.

#### 4.4.1. Actin Mediated Toxicity

Actin is ubiquitous in nature and is a highly conserved protein, which, in many organisms, represents a member of a multigene family, in which individual isoforms probably perform cell-specific functions [51]. A bundle of parallel actin filaments, cross-linked by the actin-bundling proteins (villin and fimbrin), form the core of the insect midgut microvillus [52]. Interaction of CEA with actin may have a direct influence on insect growth, development, and midgut morphology, as observed after SEM analysis. The PMM compartmentalizes the food digestion process for optimum amino acid absorption, and protects midgut cells from harmful pathogens in the RCB [24]. Degradation of PMM by CEA can therefore, significantly alter digestive process and immunity of the insect. Additionally, in the model insect *Drosophila,* binding of capping protein to actin is an essential perquisite for larval development [53]. Hence, the binding of CEA to actin might also be capable of inducing larval mortality, by hindering the binding of capping, or similar functional proteins to actin in the RCB.

#### 4.4.2. ATP Synthase Mediated Toxicity

Studies have shown that many synthetic insecticides like Allethrin, DDT, 2, 4-D, *etc*, target ATPase for inducing insect mortality [54,55]. In the present study, the ATP synthase was found to be targeted by plant lectin (CEA) for exerting entomotoxicity. Thus, targeting ATP synthase seems to be a pre-existing adaptive strategy of plants against pests. Interestingly, vacuolar ATP synthase pumps of the RCB specially regulate the K^+^ gradient formation across the midgut membrane, which is considered as the primary driving force behind active amino acid absorption [56]. Therefore, inhibition of ATP synthase activity due to CEA binding is capable of directly inhibiting the amino acid absorption by midgut epithelial cells, and finally contributing to growth retardation and death of the target insect.

#### 4.4.3. Cytochrome P450 Mediated Toxicity

In response to plant defenses, insects have developed sophisticated machinery to evade the toxic effect of insecticides or other toxins from both plant and animal origin. An increase in insecticide detoxification by Cyt P450 and other enzymes is a common mechanism of insecticide resistance in insects [57,58]. Therefore, reducing the expression of detoxifying enzymes that are involved in detoxification process of different allelochemicals can be an effective IPM strategy. The present finding suggests that CEA might follow the aforementioned route for controlling RCB by interacting with Cyt P450. Interestingly, Cyt P450s were reported to play a fundamental role in the cotton bollworm by assisting the feeder in tolerating higher concentration of toxic metabolites of cotton seeds, namely gossypol, after feeding [59]. Thus, considering the role of Cyt P450 in other cotton pests, the binding of CEA to Cyt P450 might increase RCB venerability to gossypol and other plant-derived toxic components and consequently exhibits entomotoxicity.

## 5. Conclusions

This study assessed the insecticidal efficacy of CEA against RCB and throws light on its potential agronomical applications. Since gut is the principal interface between the insect and the environment, understanding the mechanism of action of plant derived insecticidal proteins like CEA might also contribute to elucidating the co-evolutionary arms race between plants and insects. The interaction of CEA with RCB gut induces entomotoxicity by degradation of the PMM. Further identification of CEA binding partners such as actin, ATP synthase and Cyt p450 from RCB midgut unveils putative candidate proteins responsible for the CEA toxicity and also adds to the current understanding of the mechanism of entomotoxicity of monocot mannose binding lectins. RNAi mediated gene function analysis of these identified candidate proteins in the future would help to expand the repertoire of potential IPM strategies in the development of superior insecticidal candidates against sap sucking hemipterans.

## Supplementary Files

Supplementary File 1

## Abbreviations

ASAL: *Allium sativum* leaf agglutinin;
BBMV: Brush border membrane vesicle;
Bt: *Bacillus thuringiensis*;
CEA: *Colocasia esculenta* tuber agglutinin;
GNA: *Galanthus nivalis* agglutinin;
IPM: Insect pest management;
MALDI: Matrix-assisted laser desorption ionization;
MMBL: Monocot mannose binding lectin;
PMF: Peptide mass fingerprinting;
PMM: Perimicrovillar membrane;
RCB: Red cotton bug;
TOF: Time of flight
