# Supplementary material for: Molecular Mechanism Underlying the Entomotoxic Effect of Colocasia esculenta Tuber Agglutinin against Dysdercus cingulatus
Source: Insects. 2015 Oct 7;6(4):827–46. doi: 10.3390/insects6040827 (PMC4693173; doi:10.3390/insects6040827)
Supplement: Supplementary File 1 [file insects-06-00827-s001.pdf]

Supplemental Materials

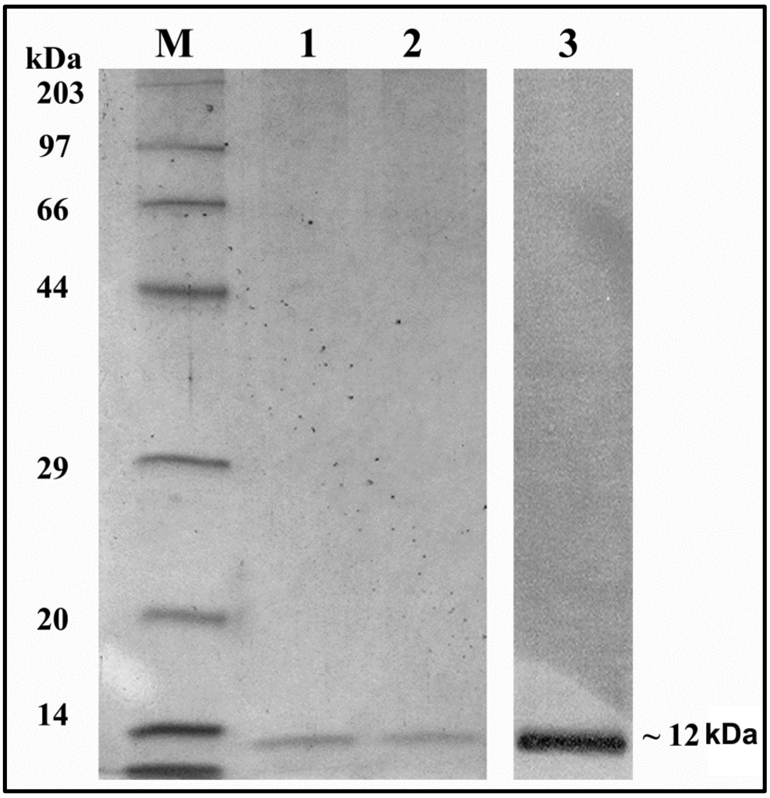

Figure S1. Purification of native CEA. SDS PAGE (15%) profile of purified fractions of native CEA. Panel 1: Lane M: Standard protein molecular weight marker; Lane 1& 2: Purified CEA fraction; Lanes 3: CEA showing ~12 kDa band after western blot analysis with anti-CEA antibody.

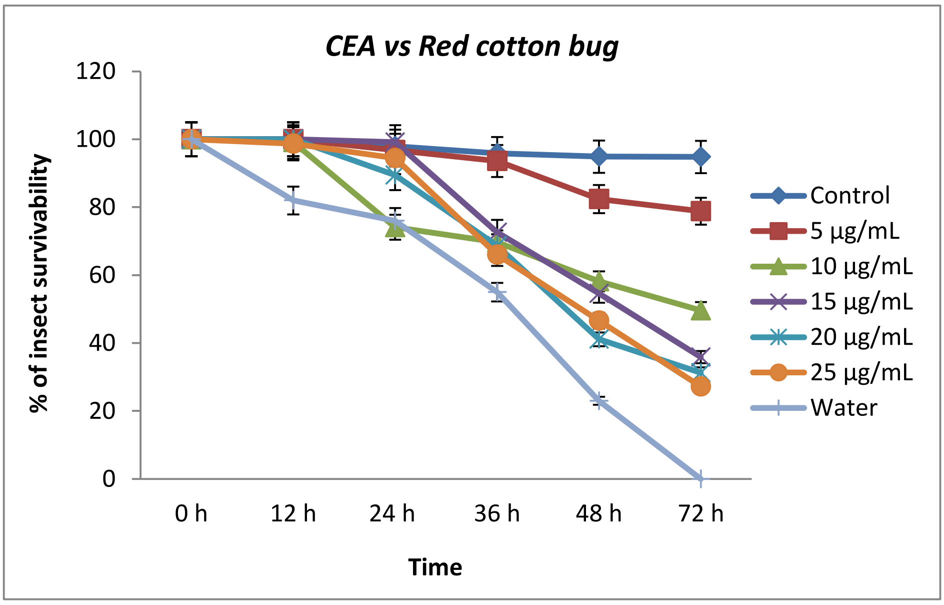

Figure S2. Insect bioassay in CEA supplemented artificial diet on second instar nymphs of red cotton bug. Graph demonstrating percentage of insect survivability at different concentrations of CEA (0, 5, 10, 15, 20, 25 µg/mL) recorded over 72 h.

Table S1. Toxicity assay of native CEA determined against *Dysdercus cingulatus*.

Target insect	LC50 Value (μg/mL)	Fiducial limit LC50 Value (μg/mL)		SE of slope	Regression equation (Y)	χ^2 value	df
		Lower	Upper				
Dysdercus cingulatus (Red cotton bug)	15.64	14.77	16.58	0.268	2.08 + 2.440x	0.358	3

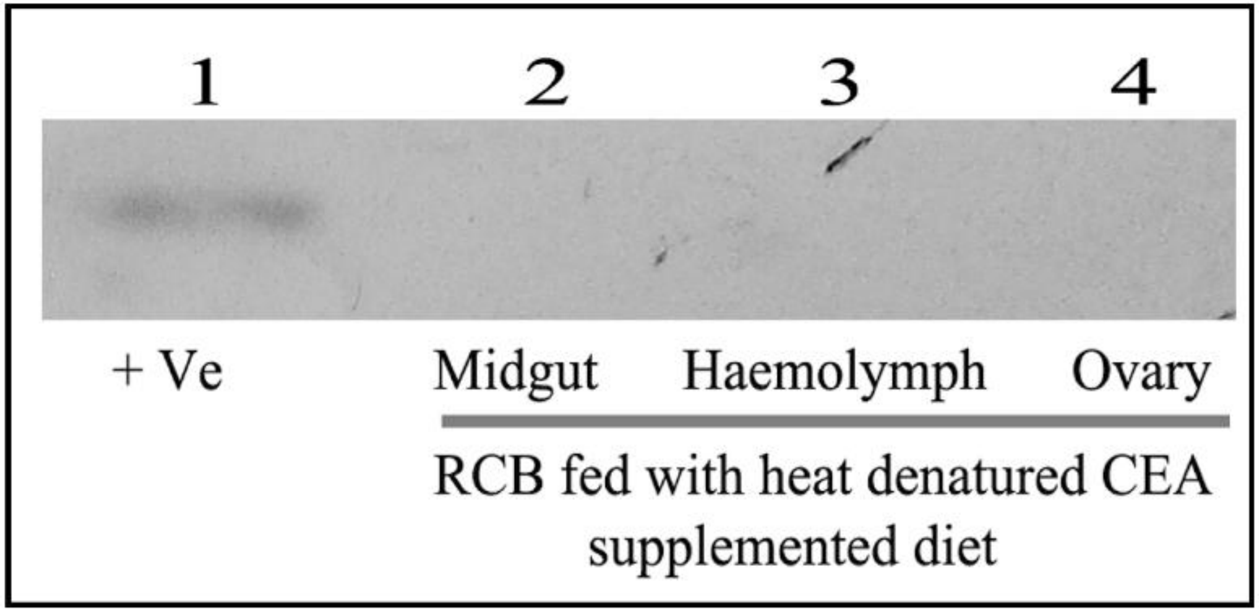

Figure S3. Western blotting with the midgut, haemolymph and ovary of *D. cingulatus* nymph fed with heat denatured CEA supplemented diet. Lane 1: CEA (0.5 μg), positive control. Lane 2, 3 & 4: total protein extract from heat denatured CEA fed RCB ovary, haemolymph and midgut showing no CEA band at ~12 kDa.

Spot 1 : Result obtained by PMF and MS/MS analysis.

Mascot Search Results

Protein View

Match to: **Q5XUA1_9HEMI** Score: **169** Expect: **1.4e-09**
Putative mitochondrial ATP synthase alpha subunit.- Toxoptera citricida (brown citrus aphid).

Taxonomy: Toxoptera citricida

Nominal mass (M_r): **599 7;** Calculated pI value: **9.14**

Fixed modifications: Carbamidomethyl (C)
Variable modifications: Oxidation (M)
Cleavage by Trypsin: cuts C-term side of KR unless next residue is P
Number of mass values searched: **28**
Number of mass values matched: **14**
Sequence Coverage: **27%**

Matched peptides shown in **Bold Red**

```
  1 MANLSLRLAT KIGKQLNNTL PQISKASWTT SKLISRSLHI TCSARAAEIS
 51 SILEERILGA PPKADLEETG RVLSIGDGIA RVYGLKNVQA EEMVEFSSGL
101 KGMALNLEPD NVGVVVFGND KLIKEGDVVK RTGAIVDVPV GEDLLGRVVD
151 ALGNTIDGKG PLTSKLRYRV GIKAPGIIPR VSVREPMQTG IKAVDSLVPI
201 GRGQRELIIG DRQTGKTALA IDTIINQKRF NDAQDEKKKL YCIYVAIGQK
251 RSTVAQIVKR LTDTGAIKYT IIVSATASDA APLQYLAPYS GCAMGEFFRD
301 NGKHALIIYD DLSKQAVAYR QMSLLLRRPP GREAYPGDVF YLHSRLLERA
351 AKMSETLGGG SLTALPVIET QAGDVSAYIP TNVISITDGQ IFLETELFYK
401 GVRPAINVGL SVSRVGSAAQ TKAMKQVAGS MKLELAQYRE VAAFAQFGSD
451 LDAATQQLLN RGVRLTELLK QGQYVPMAIE EQVAVVYCGV RGFLDKMEPS
501 KITTFEKEFL QHIKTSEKSL LESIAKEGKI TDETDAKLKS VVTNFLASFN
551 A
```

Mascot Score Histogram

Protein score is -10*Log(P), where P is the probability that the observed match is a random event
Protein scores greater than 64 are significant (p<0.05).

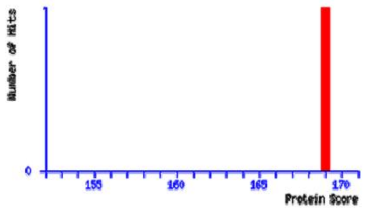

Sort Peptides By ◉ Residue Number ○ Increasing Mass ○ Decreasing Mass

Start - End	Observed	Mr(expt)	Mr(calc)	ppm	Miss	Sequence
72 - 81	1000.5503	999.5430	999.5713	-28	0	R.VLSIGDGIAR.V
87 - 101	1667.7701	1666.7628	1666.7872	-15	0	K.NVQAEEMVEFSSGLK.G
87 - 101	1683.7769	1682.7696	1682.7821	-7	0	K.NVQAEEMVEFSSGLK.G Oxidation (M)
102 - 121	2088.0239	2087.0166	2087.0358	-9	0	K.GMALNLEPDNVGVVVFGNDK.L
102 - 121	2104.0110	2103.0037	2103.0307	-13	0	K.GMALNLEPDNVGVVVFGNDK.L Oxidation (M)
132 - 147	1610.8644	1609.8571	1609.8676	-6	0	R.TGAIVDVPVGEDLLGR.V
193 - 202	1026.5698	1025.5626	1025.5869	-24	0	K.AVDSLVPIGR.G
206 - 212	815.4378	814.4305	814.4548	-30	0	R.ELIIGDR.Q
217 - 229	1456.7938	1455.7865	1455.8409	-37	1	K.TALAIDTIINQKR.F
230 - 237	966.4014	965.3941	965.4090	-15	0	R.FNDAQDEK.K
304 - 314	1287.6573	1286.6500	1286.6870	-29	0	K.HALIIYDDLSK.Q
433 - 439	892.4581	891.4508	891.4814	-34	0	K.LELAQYR.E
440 - 461	2365.1598	2364.1525	2364.1710	-8	0	R.EVAAFAQFGSDLDAATQQLLNR.G
497 - 507	1310.5643	1309.5570	1309.6588	-78	1	K.MEPSKITTFEK.E

No match to: 1051.6215, 1062.5432, 1165.6451, 1171.6176, 1177.4442, 1203.5978, 1348.5940, 1412.7696, 1427.7526, 1438.7249, 1448.8421, 1475.7308, 1501.7445, 1606.79·

MS /MS matched peptides :

Start - End	Observed	Mr(expt)	Mr(calc)	ppm	Miss	Sequence	
217 - 229	1456.7910	1455.7837	1455.8409	-39	1	K.TALAIDTIINQKR.F	(Ions score 127)
132 - 147	1610.8490	1609.8417	1609.8676	-16	0	R.TGAIVDVPVGEDLLGR.V	(Ions score 105)

Figure S4. *Cont.*

Spot 2: Result obtained by PMF analysis

Mascot Search Results

Protein View

Match to: **Q1HPZ3_BOMMO** Score: **87** Expect: **0.00029**
Mitochondrial inorganic phosphate carrier.- Bombyx mori (Silk moth).

Nominal mass (M_r): **35464;** Calculated pI value: **9.33**

Taxonomy: Bombyx mori

Fixed modifications: Carbamidomethyl (C)
Variable modifications: Oxidation (M)
Cleavage by Trypsin: cuts C-term side of KR unless next residue is P
Number of mass values searched: **16**
Number of mass values matched: **6**
Sequence Coverage: **20%**

Matched peptides shown in **Bold Red**

```
  1 MSKKPKEEFS CEMYSNKYFM LCAYGGACAC GITHTFVTPL DLVKCRLQVD
 51 PAKYKSIFKG FGISIKEGGA ANLVKGWAPT LIGYSMQGSS KFAGYEYFKY
101 KFSKMVDEES AYLYRTYLYL AAAASAECIA DIFLSPFEAT KVRIQTTPGY
151 TSKMRKAMPH MLATEGFGVF YRGLVPLWGR QIPYTMMKFA SFEKTLEYLY
201 ENVVPKPRDQ CTKIEQLLVT FTAGYIAGVL CAIVSHPADT IVSKLNKDPG
251 ASIGGIIAEV GPMGIWRGLV ARIIMMGTLT GLQGFIYDGF KVKMGMPRPP
301 PAEMPESLRK KLAAKK
```

Mascot Score Histogram

Protein score is -10*Log(P), where P is the probability that the observed match is a random event.
Protein scores greater than 64 are significant (p<0.05).

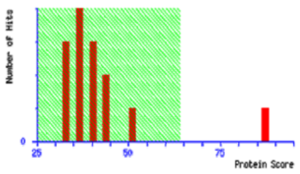

Sort Peptides By ◉ Residue Number ○ Increasing Mass ○ Decreasing Mass

Start - End	Observed	Mr(expt)	Mr(calc)	ppm	Miss	Sequence
45 - 53	1086.5314	1085.5241	1085.5652	-38	1	K.CRLQVDPAK.Y
92 - 101	1315.6565	1314.6493	1314.6284	16	1	K.FAGYEYFKYK.F
268 - 272	515.3260	514.3187	514.3227	-8	0	R.GLVAR.I
268 - 291	2617.1801	2616.1729	2616.3808	-79	1	R.GLVARIIMMGTLTGLQGFIYDGFK.V Oxidation (M)
294 - 310	1956.0232	1955.0159	1954.9427	37	1	K.MGMPRPPPAEMPESLRK.K 2 Oxidation (M)
312 - 316	530.3346	529.3273	529.3587	-59	1	K.LAAKK.-

No match to: 675.3749, 927.5311, 968.4709, 1079.5317, 1289.6118, 1501.7418,1602.7943, 1756.8645, 1888.8736, 1994.030

Figure S4. *Cont.*

Spot 3: Result obtained by PMF and MS/MS analysis .

{MATRIX SCIENCE} Mascot Search Results

Protein View

Match to: **Q16E72_AEDAE** Score: **118** Expect: **2.3e-07**
Cytochrome P450.- Aedes aegypti (Yellowfever mosquito).

Nominal mass (M_r): **48360**; Calculated pI value: **6.40**

Taxonomy: Aedes aegypti

Fixed modifications: Carbamidomethyl (C)
Variable modifications: Oxidation (M)
Cleavage by Trypsin: cuts C-term side of KR unless next residue is P
Number of mass values searched: **16**
Number of mass values matched: **10**
Sequence Coverage: **23%**

Matched peptides shown in **Bold Red**

```
  1 MQQVLCCNEL YDKPFLYDFF RLGNGILTER SGERWLQARK LVNPAFNTRM
 51 LTAFLPIMDS EAKNLCDKLE PLADGNTEID IFSHLSSCTL STTFGTTMGQ
101 NAKEIPEQHD YIRNVEIFLK AVGERLVNVY YFIEPIYKLS KAYKIHDEAR
151 RICNEFTHRI VSKRRFEIQS LGEDFQQKDE YIKQHLNALD QIITMKRPDG
201 TGFSDPEVNE HLYTLIGAGT DTSALTVAYT CLYLAMYPEV QEKVLTEINQ
251 VFYSPEVEVN IENLKQLEYT EMVIKEILRL FPAGPLGARQ TMSAIELDGI
301 RIPKDQIIIF SMFTLHRRKD IWGPDPEQFD PERFRPEAIE ARHPFAYLPF
351 SGGLRNCIGH RYAMNVMRII LLRIMQKFEI QTNMKPTDLK LKFEVTLKLD
401 GPHRVWLVRR NK
```

Mascot Score Histogram

Protein score is -10*Log(P), where P is the probability that the observed match is a random event.
Protein scores greater than 64 are significant (p<0.05).

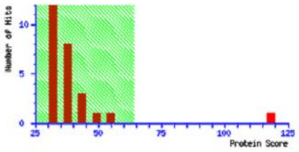

Show predicted peptides also

Sort Peptides By ◉ Residue Number ○ Increasing Mass ○ Decreasing Mass

Start - End	Observed	Mr(expt)	Mr(calc)	ppm	Miss	Sequence
41 - 63	2595.2430	2594.2357	2594.3236	-34	1	K.LVNPAFNTRMLTAFLPIMDSEAK.N Oxidation (M)
50 - 63	1566.8143	1565.8070	1565.7833	15	0	R.MLTAFLPIMDSEAK.N
50 - 63	1598.7943	1597.7871	1597.7731	9	0	R.MLTAFLPIMDSEAK.N 2 Oxidation (M)
50 - 68	2213.1385	2212.1313	2212.0578	33	1	R.MLTAFLPIMDSEAKNLCDK.L Oxidation (M)
114 - 125	1374.6747	1373.6674	1373.7667	-72	1	R.NVEIFLKAVGER.L
152 - 159	1076.4974	1075.4901	1075.4869	3	0	R.ICNEFTHR.I
276 - 279	530.3109	529.3037	529.3224	-35	0	K.EILR.L
305 - 317	1636.8240	1635.8167	1635.8443	-17	0	K.DQIIIFSMFTLHR.R Oxidation (M)
343 - 361	2199.0897	2198.0824	2198.0956	-6	1	R.HPFAYLPFSGGLRNCIGHR.Y
378 - 390	1564.7280	1563.7207	1563.7967	-49	0	K.FEIQTNMKPTDLK.L

No match to: 842.5051, 1383.6950, 1490.7330, 1645.8514, 1994.0907, 2072.4496

MS/ MS matched peptides :

Start - End	Observed	Mr(expt)	Mr(calc)	ppm	Miss	Sequence	
152 - 159	1076.4974	1075.4901	1075.4869	7	0	R.ICNEFTHR.I	(Ions score 25)
114 - 125	1374.6747	1373.6674	1373.7667	-7	1	R.NVEIFLKAVGER.L	(Ions score 42)
305 - 317	1636.8240	1635.8167	1635.8443	-13	0	K.DQIIIFSMFTLHR.R	(Ions score 58)
378 - 390	1564.7280	1563.7207	1563.7967	-29	0	K.FEIQTNMKPTDLK.L	(Ions score 57)

Figure S4. *Cont.*

Spot 4: Result obtained by Peptide mass fingerprinting (PMF).

MATRIX SCIENCE **Mascot Search Results**

Protein View

Q17LH0_AEDAE **Mass:** 15681 **Score:** 120 **Expect:** 1.4e-07

RNA-binding protein.- Aedes aegypti (Yellowfever mosquito).

Taxonomy: Aedes aegypti

Fixed modifications: Carbamidomethyl (C)
Variable modifications: Oxidation (M)
Cleavage by Trypsin: cuts C-term side of KR unless next residue is P
Number of mass values searched: **14**
Number of mass values matched: **8**
Sequence Coverage: **33%**

Matched peptides shown in **Bold Red**

1 MARYREWDLQ CKVYVGNLGS SASKHEIESA FGKYGPLRNV WVAR**NPPGFA**
51 **FVEFEDKRDA EDAVRSLDGT R**CCGTRIRVE MSSGRTRRDD RRRPRR**SYRS**
101 R**SPRR**SRSRS **RSMSRDR**DRD RRSRSGSRDR R

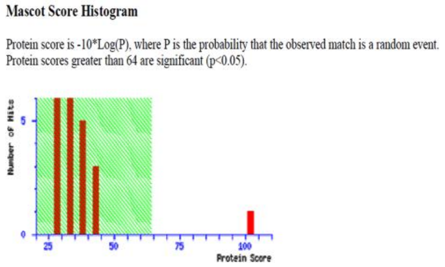

Sort Peptides By ◉ Residue Number ○ Increasing Mass ○ Decreasing Mass

Start - End	Observed	Mr(expt)	Mr(calc)	ppm	Miss	Sequence
2 - 19	1940.9443	1939.9371	1939.7710	86	0	R.NPPGFAFVEFEDK.R
20 - 29	976.4709	975.4636	975.4410	23	1	R.NPPGFAFVEFEDKR.D
59 - 65	775.3925	774.3852	774.3508	44	0	R.DAEDAVR.S
59 - 71	1404.5400	1403.5327	1403.6641	-94	1	R.DAEDAVRSLDGTR.C
97 - 99	425.2164	424.2092	424.2070	5	0	R.SYR.S
102 - 105	515.3328	514.3255	514.2976	54	1	R.SPRR.S
108 - 111	505.2419	504.2347	504.2768	-84	1	R.SRSR.S
112 - 117	751.3317	750.3244	750.3443	-26	1	R.SMSRDR.D

No match to: 2054.6976, 2068.7141, 2224.7638, 2233.7450, 2349.9611, 2810.7188

Figure S4. *Cont.*

Spot 5: Result obtained by PMF and MS/MS analysis.

{MATRIX SCIENCE} Mascot Search Results

Protein View

Match to: **Q16VS2_AEDAE** Score: **99** Expect: **0.0021**
Actin.- Aedes aegypti (Yellowfever mosquito).

Nominal mass (M_r): **41835**; Calculated pI value: **5.36**

Taxonomy: Aedes aegypti

Fixed modifications: Carbamidomethyl (C)
Variable modifications: Oxidation (M)
Cleavage by Trypsin: cuts C-term side of KR unless next residue is P
Number of mass values searched: **14**
Number of mass values matched: **9**
Sequence Coverage: **25%**

Matched peptides shown in **Bold Red**

1 MCDDDQGALV VDNGSGMCKA GFAGDDAPRA VFPSIVGRPR HQGVMVGMGQ
51 KDAYVGDEAQ SKRGILTLKY PIEHGIITNW DDMEKIWHHT FYNELRVAPE
101 EHPILLTEAP LNPKSNREKM TQIMFETFAA PAAYVAIQAV LSLYASGRTT
151 GVVLDSGDGV SHTVPIYEGY ALPHAILRMD LAGRDLTDYL MKILTERGYS
201 FTTTAEREIV RDIKEKLCYV ALDFEQEMQA AAASSSSEKS YELPDGQVIT
251 IGNERFRAPE ALFQPSFLGM EATGVHETVY NSIMRCDVDI RKDLYANSVL
301 SGGTTMYPGI ADRMQKEITS LAPSTIKIKI IAPPERKYSV WIGGSILASL
351 STFQTMWISK QEYDESGPGI VHRKCF

Mascot Score Histogram

Protein score is -10*Log(P), where P is the probability that the observed match is a random event.
Protein scores greater than 64 are significant (p<0.05).

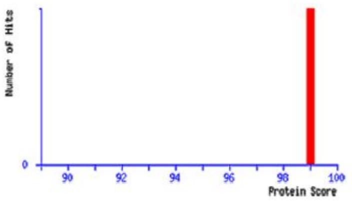

Sort Peptides By ◉ Residue Number ○ Increasing Mass ○ Decreasing Mass

Start - End	Observed	Mr(expt)	Mr(calc)	ppm	Miss	Sequence
2 - 19	1940.9443	1939.9371	1939.7710	86	0	M.CDDDQGALVVDNGSGMCK.A
20 - 29	976.4709	975.4636	975.4410	23	0	K.AGFAGDDAPR.A
41 - 51	1171.6021	1170.5949	1170.5638	27	0	R.HQGVMVGMGQK.D
41 - 51	1187.5413	1186.5341	1186.5587	-21	0	R.HQGVMVGMGQK.D Oxidation (M)
86 - 96	1515.7782	1514.7710	1514.7419	19	0	K.IWHHTFYNELR.V
97 - 114	1968.1232	1967.1159	1967.0727	22	0	R.VAPEEHPILLTEAPLNPK.S
198 - 207	1132.5647	1131.5574	1131.5197	33	0	R.GYSFTTTAER.E
240 - 255	1790.9320	1789.9247	1789.8846	22	0	K.SYELPDGQVITIGNER.F
361 - 373	1486.8300	1485.8227	1485.6848	93	0	K.QEYDESGPGIVHR.K

No match to: 1774.8866, 1804.9459, 1869.0288, 2215.0998, 2231.0888

MS /MS matched peptides :

Start - End	Observed	Mr(expt)	Mr(calc)	ppm	Miss	Sequence	
240 - 255	1791.0898	1790.0825	1789.8846	11	0	K.SYELPDGQVITIGNER.F	(Ions score 183)
361 - 373	1486.8300	1485.8227	1485.6848	9[illegible]	0	K.QEYDESGPGIVHR.K	(Ions score 129)

Figure S4. *Cont.*

Spot 6: Result obtained after Pertide mass fingerprinting (PMF)

MATRIX SCIENCE Mascot Search Results

Protein View

```
Match to: gi|170040984 Score: 106 Expect: 2.2e-05
26S proteasome non-ATPase regulatory subunit 1 [Culex quinquefasciatus]

Nominal mass (Mr): 112988; Calculated pI value: 5.50

Taxonomy: Culex quinquefasciatus

Fixed modifications: Carbamidomethyl (C)
Variable modifications: Oxidation (M)
Cleavage by Trypsin: cuts C-term side of KR unless next residue is P
Number of mass values searched: 14
Number of mass values matched: 10
Sequence Coverage: 14%

Matched peptides shown in Bold Red

   1 MKLNITSAAG IICLLDEPIQ ELKVFALKKL DTIVDEFWPE ISEAVEKIEI
  51 LHEDKGFQQH DLAALVASKV YYHLGSFEDS LTYALGAGDL FDVNARNEYV
 101 DTIIAKCIDH YTQLRVQLAE NPAKAKPIDA RLEAIVNRMI QRCLDDGQYR
 151 QALGIALETR RMDVVESSIM KADDIAGMLA YAFQVTMSLI QNRAFRNTVL
 201 RCLVGLYRNL GVPDYVNMCQ CLIFLEDPLA VAELLDNLTK GGEHSVLMAY
 251 QIAFDLYESA TQQFLGQVLQ ALKATAPIPS ALISNLKPQG TNAAGAAAAP
 301 EVKQEPKEVG EESEGGDVKI ERTVDSLNES EKTHQANIEK LAGILSGEIT
 351 IDLQLQFLIR SNHADLQILR ATKEAVRVSI CHTATVIANA FMHSGTTSDQ
 401 FLRDNLEWLA RATNWAKLTA TASLGVIHRG HETESLALMQ SYLPKESGPS
 451 SGYSEGGGLY ALGLIHANHG ANIIDYLLQQ LKDAQNENVR HGGCLGLGLA
 501 AMGTHRQDVY EQLKFNLYQD DAVTGEAAGI AMGMVMLGSK HAPAIEDMVA
 551 YAQETQHEKI LRGLAVGISL TMYARLEEAD SLVTSLSNDK DPVLRRSGMY
 601 TIAMAYCGTG NNQAIRKLLH VAVSDVNDDV RRAAVTAIGF ILFRTPEQCP
 651 SVVSLLAESY NPHVRYGAAM ALGIACAGTG LREAIALLEP MAKFDPVNFV
 701 RQGALIASAM ILIQQTDQTC PKVTFFRQLY TQVITNKHED VMAKYGAILA
 751 QGIIDAGGRN VTVSLQSRTG HTNLQAVVGM LVFTQYWYWF PLAHCLSLAF
 801 TPTCLIALNS DLKMPKVDLK SAARPSLYSY PAPLEEKKRE ERDKVATAVL
 851 SLAARAKRRT GDKKKDNAKD ADSKMDVDDE ASATPPKDDL SATVKAKEEA
 901 KTTPVKEDKK KEAKPKDAAT PSPAPAEETP ANAAAATAAE KKTPKEPEPS
 951 FEILHNPARV MRAQLKVISI AEGTPFTPLK DVAIGGIVMM QHNAAAGEQV
1001 LVERVTAYGP KNDDLKEPEA PEPFEYIED
```

Mascot Score Histogram

Ions score is -10*Log(P), where P is the probability that the observed match is a random event. Individual ions scores > 45 indicate identity or extensive homology (p<0.05). Protein scores are derived from ions scores as a non-probabilistic basis for ranking protein hits.

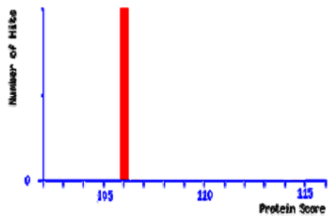

Sort Peptides By ◉ Residue Number ○ Increasing Mass ○ Decreasing Mass

Start – End	Observed	Mr(expt)	Mr(calc)	ppm	Miss	Sequence
125 – 138	1565.8692	1564.8619	1564.9049	-27	1	K.AKPIDARLEAIVNR.M
151 – 161	1227.7015	1226.6942	1226.7095	-12	1	R.QALGIALETRR.M
161 – 171	1294.7386	1293.7313	1293.6421	69	1	R.RMDVVESSIMK.A
404 – 411	1016.5755	1015.5682	1015.5087	59	0	R.DNLEWLAR.A
515 – 540	2705.2843	2704.2771	2704.2547	8	0	K.FNLYQDDAVTGEAAGIAMGMVMLGSK.H Oxidation (M)
560 – 575	1734.0388	1733.0316	1733.0022	17	1	K.ILRGLAVGISLTMYAR.L
597 – 616	2211.1372	2210.1300	2209.9554	79	0	R.SGMYTIAMAYCGTGNNQAIR.K 2 Oxidation (M)
618 – 632	1707.8625	1706.8553	1706.9064	-30	1	K.LLHVAVSDVNDDVRR.A
666 – 682	1652.9025	1651.8952	1651.8174	47	0	R.YGAAMALGIACAGTGLR.E
1005 – 1016	1320.6957	1319.6884	1319.6721	12	1	R.VTAYGPKNDDLK.E

No match to: 850.5569, 956.5536, 1945.0007, 2369.0750

Figure S4. (**Spot 1**) MALDI TOF/TOF identification of ATP synthase α subunit from brown citrus aphid. (**Spot 2**) MALDI TOF/TOF identification of mitochondrial inorganic phosphate carrier from silk moth. (**Spot 3**) MALDI TOF/TOF identification of Cyt P450 from Yellow fever mosquito. (**Spot 4**) MALDI TOF/TOF identification of RNA binding protein from Yellow fever mosquito. (**Spot 5**) MALDI TOF/TOF identification of actin from Yellow fever mosquito. (**Spot 6**) MALDI TOF/TOF identification of 26s proteasome non-ATPase regulatory subunit from *Culex* mosquito.

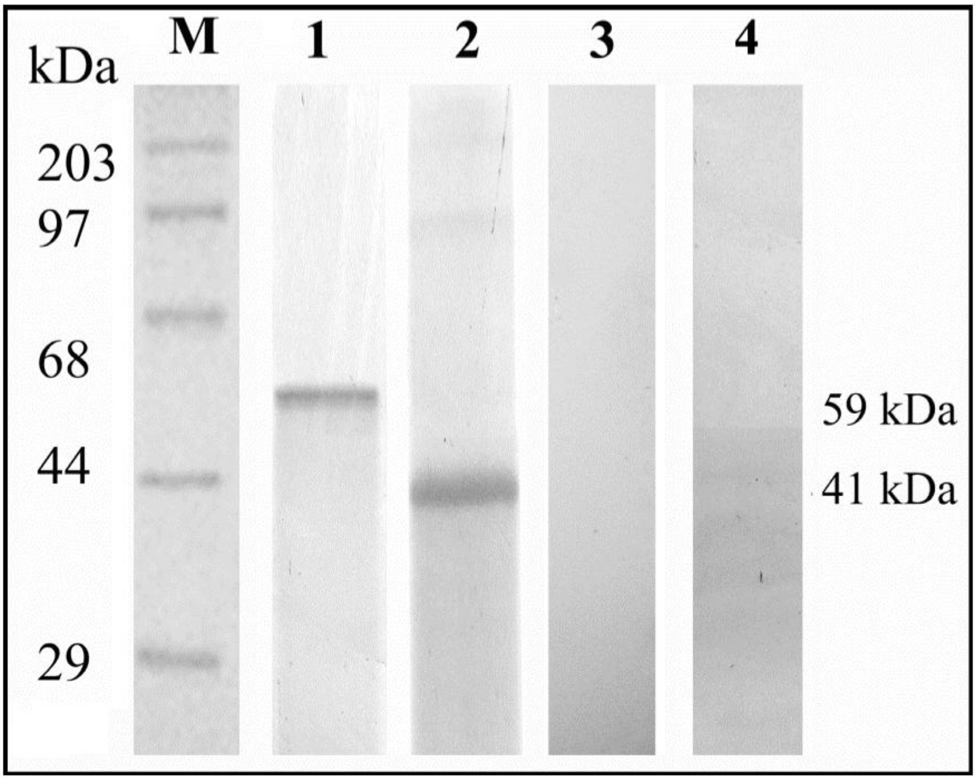

Figure S5. Immunoprecipitation and subsequent ligand blot analysis of Red cotton bug BBMV with CEA. Lane M: Standard protein molecular weight marker; Lane 1: ligand blotting with total BBMV immunoprecipitated with anti-ATP synthase antibody. Lane 2: ligand blotting with total BBMV immunoprecipitated with anti-actin antibody. Lane 3 & 4: immunoprecipitation without anti ATP synthase and anti actin antibody incubation (negative control).

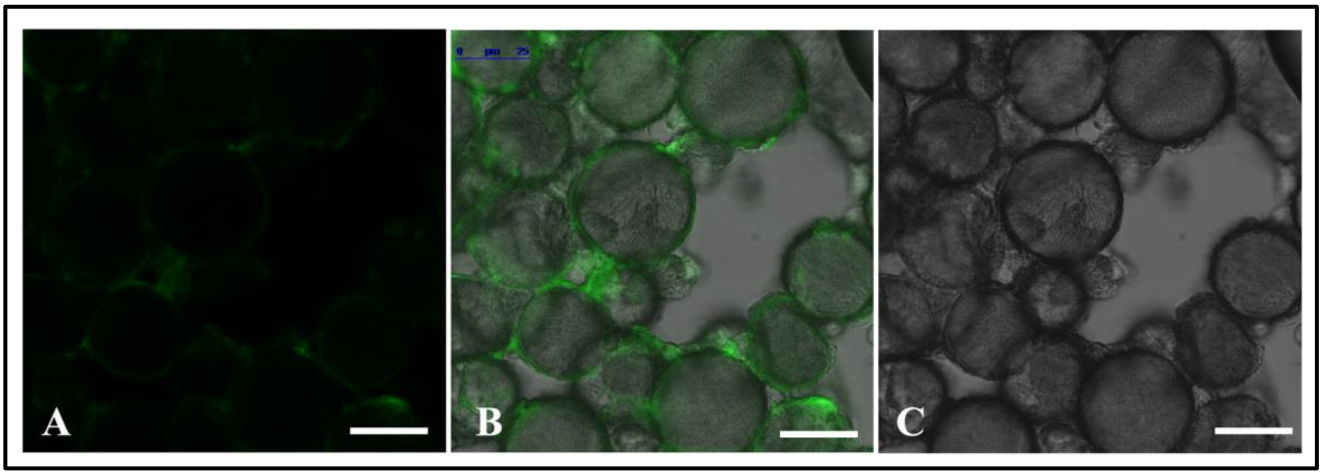

Figure S6. Confocal microscopic images showing the partial inhibition of binding of CEA to the midgut epithelial cells of *D. cingulatus* due to feeding the artificial diet supplemented with anti-actin antibody. (**A**) Fluorescence image showing significantly reduced binding of CEA to midgut epithelial cells of insects fed with anti-actin supplemented diet; (**B**) Merged image; (**C**) Phase contrast image. [Bar = 25 µm]

NetNglyco 1.0 server prediction of N glycosylation:

ATP synthase

Sequence Name: tr_Q5XUA1_Q5XUA1_TOXCI Length: 551

>tr|Q5XUA1|Q5XUA1_TOXCI ATP synthase subunit alpha OS=Toxoptera citricida PE=2 SV=1
MA**NLS**LRLATKIGKQL**NNTL**PQISKASWTTSKLISRSLHITCSARAAEISSILEERILGA
PPKADLEETGRVLSIGDGIARVYGLKNVQAEEMVEFSSGLKGMALNLEPDNVGVVVFGND
KLIKEGDVVKRTGAIVDVPVGEDLLGRVVDALGNTIDGKGPLTSKLRYRVGIKAPGIIPR
VSVREPMQTGIKAVDSLVPIGRGQRELIIGDRQTGKTALAIDTIINQKRFNDAQDEKKKL
YCIYVAIGQKRSTVAQIVKRLTDTGAIKYTIIVSATASDAAPLQYLAPYSGCAMGEFFRD
NGKHALIIYDDLSKQAVAYRQMSLLLRRPPGREAYPGDVFYLHSRLLERAAKMSETLGGG
SLTALPVIETQAGDVSAYIPTNVISITDGQIFLETELFYKGVRPAINVGLSVSRVGSAAQ
TKAMKQVAGSMKLELAQYREVAAFAQFGSDLDAATQQLLNRGVRLTELLKQGQYVPMAIE
EQVAVVYCGVRGFLDKMEPSKITTFEKEFLQHIKTSEKSLLESIAKEGKITDETDAKLKS
VVTNFLASFNA

(Threshold=0.5)

SeqName	Position	Potential	Jury agreement	N-Glyc result
tr_Q5XUA1_Q5XUA1_TOXCI	3 NLSL	0.7272	(9/9)	++
tr_Q5XUA1_Q5XUA1_TOXCI	17 NNTL	0.7161	(9/9)	++

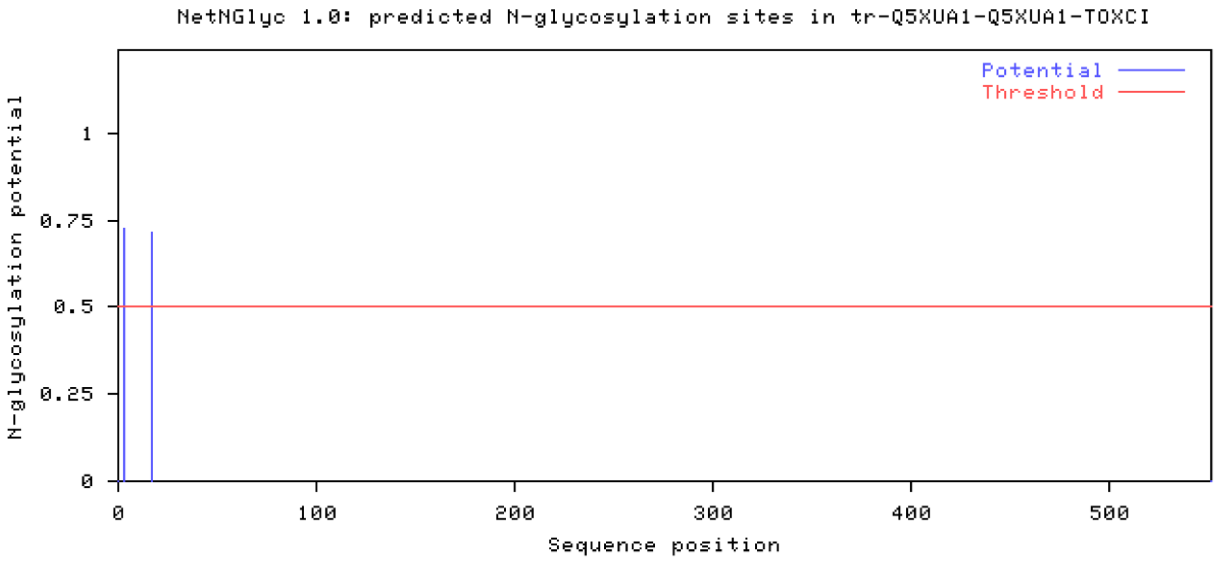

Figure S7. *Cont.*

NetCGlyc 1.0 produces neural network predictions of C-mannosylation sites

ATP synthase

```
#gff-version 2
##source-version netCglyc-1.0b
##date 2014-10-02
##Type Protein
# seqname                source        feature  start    end   score +/-  ?
# ------------------------------------------------------------------------
tr_Q5XUA1_Q5XUA1_TOXCI   netCglyc-1.0b     C-manno     28     28   0.193  .   .
# ------------------------------------------------------------------------
```

YinOYang 1.2 Prediction Results: The predictions for O-GlcNAc sites

ATP synthase

SeqName	Residue		O-GlcNAc result	Potential	Thresh. (1)	Thresh. (2)
tr_Q5XUA1_Q	24	S	++	0.5384	0.4258	0.5243
tr_Q5XUA1_Q	30	T	+	0.5384	0.4514	0.5589
tr_Q5XUA1_Q	31	S	+	0.5018	0.4657	0.5781
tr_Q5XUA1_Q	253	T	+	0.4742	0.4416	0.5457
tr_Q5XUA1_Q	270	T	+	0.6203	0.5213	0.6531
tr_Q5XUA1_Q	274	S	+	0.5187	0.5099	0.6377
tr_Q5XUA1_Q	354	S	+	0.4868	0.4398	0.5432
tr_Q5XUA1_Q	370	T	+	0.5066	0.4591	0.5692
tr_Q5XUA1_Q	381	T	+	0.5280	0.4985	0.6224
tr_Q5XUA1_Q	413	S	+	0.5276	0.4878	0.6079
tr_Q5XUA1_Q	421	T	+	0.5316	0.4408	0.5445

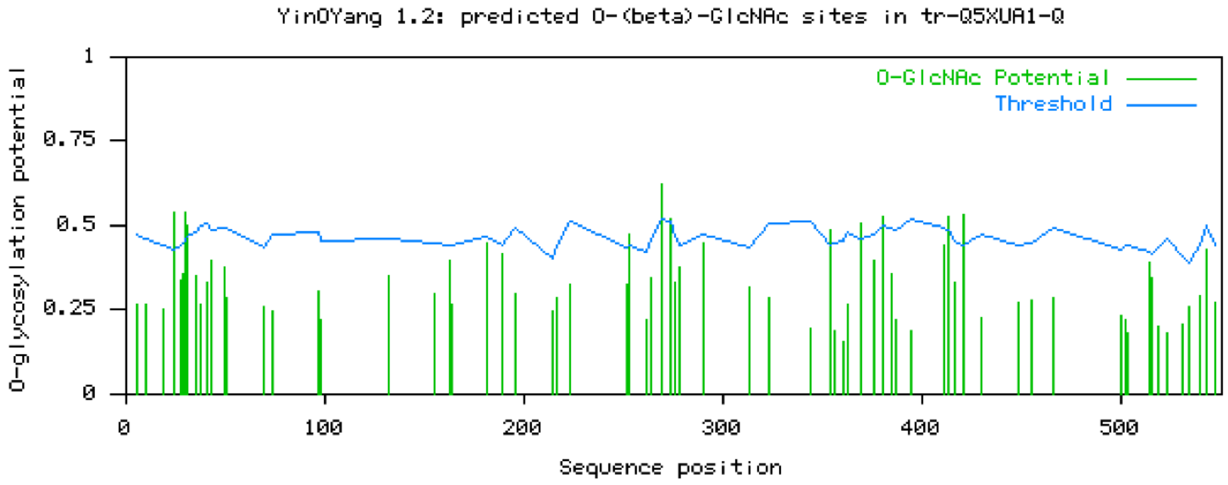

Figure S7. *Cont.*

NetNglyco 1.0 server prediction of N glycosylation:

ACTIN

>tr|Q16VS2|Q16VS2_AEDAE AAEL009451-PA OS=Aedes aegypti GN=AAEL009451 PE=3 SV=1
MCDDDQGALVVDNGSGMCKAGFAGDDAPRAVFPSIVGRPRHQGVMVGMGQKDAYVGDE
AQ
SKRGILTLKYPIEHGIITNWDDMEKIWHHTFYNELRVAPEEHPILLTEAPLNPKSNREKM
TQIMFETFAAPAAYVAIQAVLSLYASGRTTGVVLDSGDGVSHTVPIYEGYALPHAILRMD
LAGRDLTDYLMKILTERGYSFTTTAEREIVRDIKEKLCYVALDFEQEMQAAAASSSSEKS
YELPDGQVITIGNERFRAPEALFQPSFLGMEATGVHETVYNSIMRCDVDIRKDLYANSVL
SGGTTMYPGIADRMQKEITSLAPSTIKIKIIAPPERKYSVWIGGSILASLSTFQTMWISK
HEYDEGGPGIVHRKCF

(Threshold=0.5)

SeqName	Position	Potential	Jury agreement	N-Glyc result
**tr_Q16VS2_Q16VS2_AEDAE**	**13 NGSG**	**0.6679**	**(9/9)**	++

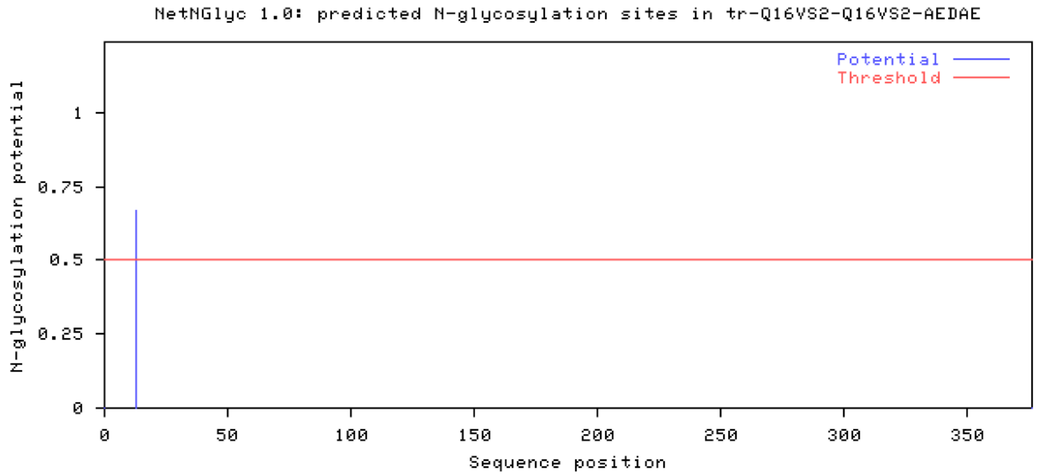

NetCGlyc 1.0 produces neural network predictions of C-mannosylation sites

Actin

##gff-version 2
##source-version netCglyc-1.0b
##date 2014-10-02
##Type Protein

# seqname	source	feature	start	end	score	+/-	?
tr_Q16VS2_Q16VS2_AEDAE	netCglyc-1.0b	C-manno	80	80	0.173	.	.
tr_Q16VS2_Q16VS2_AEDAE	netCglyc-1.0b	C-manno	87	87	0.235	.	.
tr_Q16VS2_Q16VS2_AEDAE	netCglyc-1.0b	C-manno	341	341	0.224	.	.
tr_Q16VS2_Q16VS2_AEDAE	netCglyc-1.0b	C-manno	357	357	0.154	.	.

Figure S7. *Cont.*

YinOYang 1.2 Prediction Results: The predictions for O-GlcNAc sites

Actin

SeqName	Residue		O-GlcNAc result	Potential	Thresh. (1)	Thresh. (2)
tr_Q16VS2_Q	234	S	++	0.5698	0.4073	0.4994
tr_Q16VS2_Q	235	S	+++	0.5950	0.3888	0.4745
tr_Q16VS2_Q	236	S	+++	0.5557	0.3712	0.4507
tr_Q16VS2_Q	320	S	+	0.5354	0.4416	0.5456
tr_Q16VS2_Q	325	T	+	0.4977	0.4495	0.5562

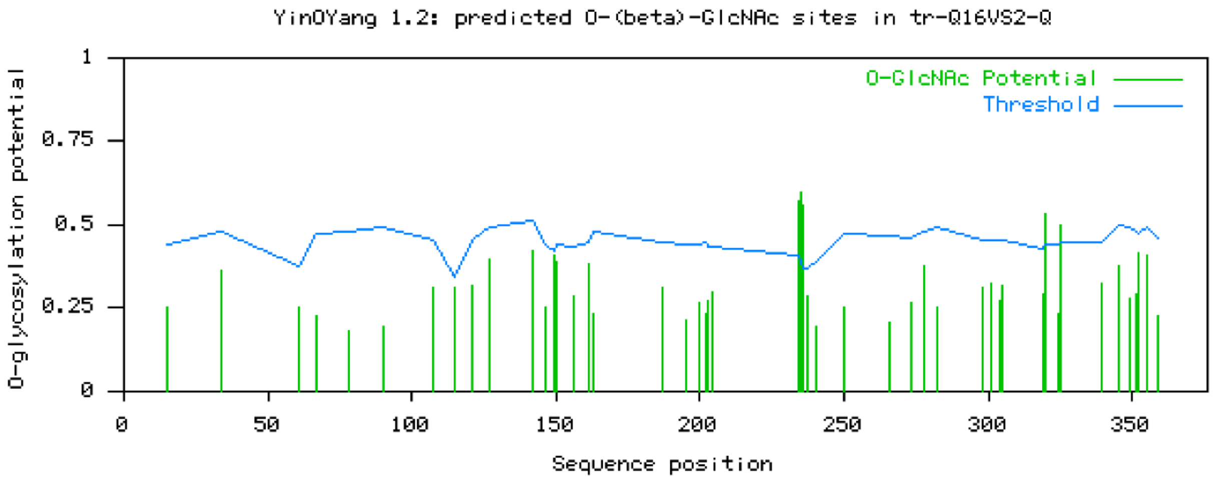

Cytocrome P450

>tr|Q16E72|Q16E72_AEDAE AAEL015361-PA OS=Aedes aegypti GN=AAEL015361 PE=3 SV=1
MQQVLCCNELYDKPFLYDFFRLGNGILTERSGERWLQARKLVNPAFNTRMLTAFLPIMDS
EAKNLCDKLEPLADGNTEIDIFSHLSSCTLSTTFGTTMGQNAKEIPEQHDYIRNVEIFLK
AVGERLVNVYYFIEPIYKLSKAYKIHDEARRICNEFTHRIVSKRRFEIQSLGEDFQQKDE
YIKQHLNALDQIITMKRPDGTGFSDPEVNEHLYTLIGAGTDTSALTVAYTCLYLAMYPEV
QEKVLTEINQVFYSPEVEVNIENLKQLEYTEMVIKEILRLFPAGPLGARQTMSAIELDGI
RIPKDQIIIFSMFTLHRRKDIWGPDPEQFDPERFRPEAIEARHPFAYLPFSGGLRNCIGH
RYAMNVMRIILLRIMQKFEIQTNMKPTDLKLKFEVTLKLDGPHRVWLVRRNK

[Note: CytP450 does not have any N-glycosylation site according to the prediction of NetNglyco 1.0 server]

Figure S7. *Cont.*

NetCGlyc 1.0 produces neural network predictions of C-mannosylation sites

Cytocrome P450

```
##gff-version 2
##source-version netCglyc-1.0b
##date 2014-10-02
##Type Protein
# seqname             source       feature  start   end   score +/-  ?
# ---------------------------------------------------------------------------
tr_Q16E72_Q16E72_AEDAE   netCglyc-1.0b      C-manno    35    35   0.166  .  .
tr_Q16E72_Q16E72_AEDAE   netCglyc-1.0b      C-manno   322   322   0.179  .  .
tr_Q16E72_Q16E72_AEDAE   netCglyc-1.0b      C-manno   406   406   0.207  .  .
# ------------
```

YinOYang 1.2 Prediction Results:The predictions for O-GlcNAc sites

Cytocrome P450

SeqName	Residue		O-GlcNAc result	Potential	Thresh. (1)	Thresh. (2)
tr_Q16E72_Q	222	T	+	0.5128	0.4363	0.5385

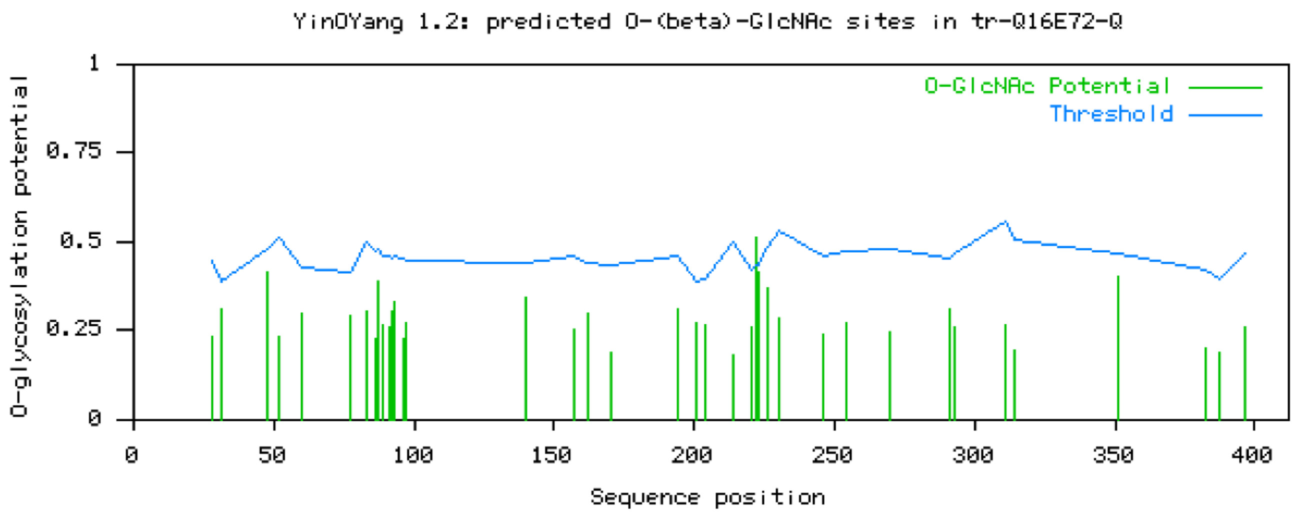

Figure S7. Glycosylation and mannosylation site prediction of putative RCB interactive partners.

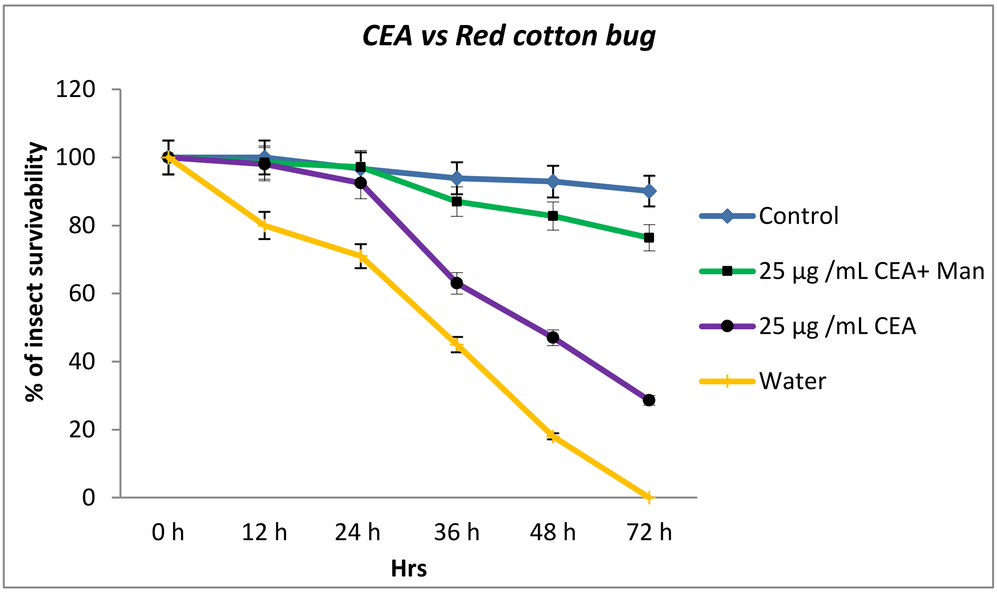

Figure S8. Insect bioassay in mannose presaturated CEA supplemented artificial diet on second instar nymphs of red cotton bug. Insects fed with or without mannose presaturated CEA (25 µg/mL) supplemented artificial diet. Insect survivability graph recorded over 72 h showing the improved survivability percentage (approximately double) when fed with CEA, presaturated with 1 M Mannose. Only diet and water served as positive and negative control, respectively.

References

1. Gupta, R. Prediction of Glycosylation Sites in Proteomes: From Post-Translational Modification to Protein Functions. Ph.D. Thesis, Center for Biological Sequence Analysis, Department of Systems Biology, Technical University of Denmark, Lyngby, Denmark, 2001.
2. Gupta, R.; Brunak, S. Prediction of glycosylation across the human proteome and the correlation to protein function. *Pac. Symp. Biocomput*. **2002**, *7*, 310–312.
3. Julenius, K. NetCglyc 1.0: Prediction of mammalian C-mannosylation sites. *Glycobiology* **2007**, *17*, 868–876.

© 2015 by the authors; licensee MDPI, Basel, Switzerland. This article is an open access article distributed under the terms and conditions of the Creative Commons Attribution license (http://creativecommons.org/licenses/by/4.0/).
